# Interpretative comments - need for harmonization? Results of the Croatian survey by the Working Group for Post-analytics

**DOI:** 10.11613/BM.2022.010901

**Published:** 2021-12-15

**Authors:** Vladimira Rimac, Sonja Podolar, Anja Jokic, Jelena Vlasic Tanaskovic, Lorena Honovic, Jasna Lenicek Krleza

**Affiliations:** 1 Working Group for Post-analytics, Croatian Society of Medical Biochemistry and Laboratory Medicine, Zagreb, Croatia; 2 Department of Transfusion Medicine and Transplantation Biology, University Hospital Centre Zagreb, Zagreb, Croatia; 3 Medical Biochemistry Laboratory, General Hospital “Dr. Tomislav Bardek”, Koprivnica, Croatia; 4 Department of Medical Biochemistry, Haematology and Coagulation with Cytology, University Hospital for Infectious Diseases “Dr. Fran Mihaljević”, Zagreb, Croatia; 5 Department of Laboratory Diagnostics, General Hospital Pula, Pula, Croatia; 6 Department of Laboratory Diagnostics, Children’s Hospital Zagreb, Zagreb, Croatia; 7 Croatian Centre for Quality Assessment in Laboratory Medicine (CROQALM), Croatian Society of Medical Biochemistry and Laboratory Medicine, Zagreb, Croatia

**Keywords:** post-analytical phase, clinical laboratory, interpretative comments, questionnaire

## Abstract

**Introduction:**

Interpretation of laboratory test results is a complex post-analytical activity that requires not only understanding of the clinical significance of laboratory results but also the analytical phase of laboratory work. The aims of this study were to determine: 1) the general opinion of Croatian medical biochemistry laboratories (MBLs) about the importance of interpretative comments on laboratory test reports, and 2) to find out whether harmonization of interpretative comments is needed.

**Materials and methods:**

This retrospective study was designed as a survey by the Working Group for Post-analytics as part of national External Quality Assessment (EQA) program. All 195 MBLs participating in the national EQA scheme, were invited to participate in the survey. Results are reported as percentages of the total number of survey participants.

**Results:**

Out of 195 MBLs, 162 participated in the survey (83%). Among them 59% MBLs implemented test result comments in routine according to national recommendations. The majority of laboratories (92%) state that interpretative comments added value to the laboratory reports, and a substantial part (72%) does not have feedback from physicians on their significance. Although physicians and patients ask for expert opinion, participants stated that the lack of interest of physicians (64%) as well as the inability to access patient’s medical record (62%) affects the quality of expert opinion.

**Conclusion:**

Although most participants state that they use interpretative comments and provide expert opinions regarding test results, results of the present study indicate that harmonization for interpretative comments is needed.

## Introduction

Nowadays, when the quality of the analytical phase of laboratory work has reached the highest level, laboratories should focus more on the extra-analytical phase of the total testing process. The post-analytical phase includes activities related to reporting of laboratory test results as well as activities that are performed before the results are communicated to the physicians. Interpretation of laboratory test results is a complex post-analytical activity that requires not only understanding of the clinical significance of the results but also the analytical part of the laboratory work and possible pre-analytical influence on test results, as well as patient’s clinical condition. In order to provide the best possible understanding of test results, it is necessary to consider how laboratory professionals can contribute to their interpretation, in order to provide physicians better understanding of the obtained results (*1*-*3*). Harmonization of this part of the post-analytical phase would minimize the existing differences between laboratories in the interpretation of test results and in that way would certainly contribute to the quality of interpretative comments (*4*). Although there are no universal guidelines regarding the use of interpretative comments, the International Organization for Standardization 15189 standard in its section 5.8.3 states that the laboratory report should include them where appropriate, which indicates the importance of using interpretative comments in routine work (*5*).

The Working Group for Post-analytics of the Croatian Society for Medical Biochemistry and Laboratory Medicine (CSMBLM) in cooperation with the Croatian Center for Quality Assessment in Laboratory Medicine (CROQALM) conducted a survey among Croatian medical biochemistry laboratories (MBLs) related to the implementation of “Post-analytical laboratory work: national recommendations from the Working Group for Post-analytics on behalf of the Croatian Society of Medical Biochemistry and Laboratory Medicine” (*6*).

The aims of this study were to determine: 1) the general opinion of MBLs on the importance of interpretative comments on laboratory test reports, and 2) to find out whether harmonization of interpretative comments is needed.

## Material and methods

### Methods

This retrospective study was designed as a survey by the Working Group for Post-analytics in cooperation with the CROQALM. In March 2021, an online questionnaire was distributed as part of the national external quality assessment (EQA) scheme and participation in it was voluntary.

As part of the EQA scheme, Module 10 entitled „Post-analytical phase of laboratory testing” contained a survey comprising 10 questions. The bases of the survey were interpretative comments and the content of the laboratory report.

### Data analysis

Results are reported as percentages of the total number of participants in the survey. Data was collected through the SurveyMonkey application, archived and processed in Microsoft Excel 2010 program (Microsoft, Redmond, USA).

## Results

Out of 195 MBLs, 162 participated in the survey, yielding a response rate of 83%. Among all laboratories that participated in survey, 59% implemented test result comments in routine work according to national recommendations, most commonly comprising those related to the pre-analytical phase. As for interpretative comments, 43% laboratories use only predefined comments on the report, while 29% laboratories do not use interpretative comments at all. The majority of laboratories (92%) state that interpretative comments added value to the laboratory reports. At the same time, a substantial percentage (72%) does not have feedback from physicians on their significance. Questions related to the expert opinion and releasing laboratory reports, as well as all survey questions and answers are presented in the Table 1.

**Table 1:** Results of national survey on interpretative comments and importance of communication with physicians regarding the results of laboratory tests in Croatia

**Question**	**Answers**	**N (%)**
1. What is the type of your institution?	Primary health care	106 (65)
	Secondary health care	42 (26)
	Tertiary health care	14 (9)
2. In what form do you report laboratory results?	Electronic laboratory report through the hospital information system or through the Central Health Information System of the Republic of Croatia (CEZIH)	105 (64)
	Printed laboratory report	19 (12)
	Protected form of laboratory report (*e.g.*, pdf) sent by e-mail to the patient	19 (12)
	Protected form of laboratory report (*e.g.*, pdf) sent by e-mail to the physician	19 (12)
3. Does your laboratory use the comments on laboratory reports recommended by the National recommendations from the Working Group for Post-analytics	Yes, all comments	96 (59)
	Yes, some comments	66 (41)
	No	0 (0)
4. For which phase of laboratory process laboratory use own comments other than those defined in the National recommendations (multiple-choice answers)	Pre-analytical phase	96 (60)
	Analytical phase	63 (39)
	Post-analytical phase	60 (38)
	We do not have our own comments	39 (24)
5. Do you have predefined interpretative comments for the tests included in CROQALM	Yes, we have predefined interpretative comments	70 (43)
	Yes, in addition to pre-defined interpretative comments, we also have a descriptive comment where applicable (*e.g.*, recommendation for reflective testing)	45 (28)
	We do not use interpretative comments	47 (29)
6. Do you think interpretative comments give added value to a laboratory report?	Yes	32 (20)
	Yes, but I do not have feedback from the physician	114 (72)
	No	13 (8)
7. Who ask for an expert opinion in the field of medical biochemistry and laboratory medicine?	Physicians	77 (49)
	Nurses	4 (2)
	Patients	77(49)
8. Expert opinion in your laboratory usually includes:	Advice on the pre-analytical phase: sample type, transport conditions or criteria for non-acceptance of samples	78 (49)
	Counseling on specific clinical cases	13 (9)
	Interpretation of obtained results through explanation of expected and unexpected results	64 (40)
	Participation in professional meetings with physician related to the effective use of laboratory services	4 (2)
9. Which of the following do you think has the greatest impact on the quality of expert opinion in the case of laboratory professionals? (multiple-choice answers)	Limited access to patient’s medical record	100 (62)
	Insufficient professional knowledge	15 (9)
	Lack of communication	19 (12)
	Unavailability/lack of interest of physician	104 (64)
	Lack of time	65 (40)
10. Which of the following do you think would make it easier to provide expert opinion to laboratory experts? (multiple-choice answers)	Access to the patient’s medical record	93 (58)
	Existence of databases of laboratory tests in the Croatian language, or national guidelines and algorithms	100 (62)
	Presentations of specific cases in literature or at professional meetings	104 (65)
	Communication skills education	43 (27)
	Participation in external quality control involving interpretive comments	55 (34)
CROQALM - Croatian Center for Quality Assessment in Laboratory Medicine. N - number of answers to each question option.		

## Discussion

This survey was focused on the practice of Croatian MBLs on providing interpretative comments and laboratory professional’s opinion in the field of medical biochemistry and laboratory medicine.

The study revealed that all participants use comments on laboratory reports that are most commonly derived from the national recommendations. Those include comments related to sample quality (pre-analytical phase), the analytical method (analytical phase), as well as comments related to post-analytics, *e.g.* remarks indicating analysis performed at physician’s request from a pre-analytically inadequate sample. However, specific laboratory settings require comments that differ from the proposed ones in the recommendations, and those are most often referred to the pre-analytical phase, which laboratory professionals use depending on the specific requirements of their laboratory process. Laboratory professionals from Croatian MBLs consider that implementation of interpretative comments into routine work is useful and gives added value to the laboratory report, thus contributing to the easier understanding of numerical laboratory test results. Laboratory professionals from other countries equally share this opinion. Specifically, Buoro *et al.*, and Plebani reveal in their studies that interpretative comments establish a positive relationship between laboratory and the clinic, further stating that interpretation of test results reduces time to diagnosis, prevents misdiagnosis and reduces the number of laboratory tests performed (*4*, *7*).

As in all phases of laboratory work, informatization has contributed to the progress of the work process in the post-analytical phase. Autovalidation and electronic reporting of laboratory results are major benefits of informatization (*6*, *8*). This was also confirmed in this survey. The vast majority of participants use either hospital information system or/and Central Health Information System of the Republic of Croatia for reporting of laboratory test results. In addition, laboratory results are commonly sent by e-mail. Regardless of informatization and optimization of the post-analytical phase, there are still areas that need to be improved or harmonized.

Most of laboratories included in this survey stated that they use interpretative comments, however, some use predefined, while other descriptive comments, depending on their applicability. Prior to the publication of the national recommendations for the post-analytical phase, 3-35% of Croatian MBLs did not use interpretative comments at all (*6*). The recommendations contributed to implementation of interpretative comments, but there is still no uniformity in their use in routine laboratory work. Such heterogeneity can lead to unclear interpretations of laboratory test results (*9*). To prevent or reduce the incorrect use of interpretative comments, survey participants state that the existence of a database of laboratory tests or participation in EQA schemes and education would make it easier to give an expert opinion and more accurate interpretation of test results. This was equally shown in the study by Ajzner, stating that improved quality can be achieved by education, availability of evidence-based guidelines or established EQA programs (*1*). Furthermore, Vasikaran *et al.* in their study report that analysis of data from EQA programs may ensure better quality of interpretative comments (*10*). Universal recommendations could provide harmonization of interpretative comments, as attested by Buoro and colleagues in recommendations for interpretative comments in laboratory haematology (*4*).

Participants in our survey state that physicians and nurses, but sometimes also patients, seek laboratory expert opinion, most often related to the pre-analytical phase or interpretation of obtained results through explanation of expected or unexpected test results. Laboratory professionals also state that the non-availability of patient’s medical record or limited communication with physicians, as well as insufficient professional knowledge or scarce feedback from physicians has a major impact on the quality of expert opinion. In their study, Huang *et al.* also emphasized similar pitfalls for a more successful interpretation of laboratory test results. They stated that generally insufficient clinical information and inadequate expertise in the subspecialty area of laboratory medicine and clinical knowledge are factors that influence the quality of interpretative comments (*3*). Dana *et al.* emphasized the problem in communication between laboratory and clinic, indicating physicians’ unavailability when laboratory staff wants to report an unexpected laboratory test result can lead to a loss of time in the laboratory due to repeated calls to the clinic, but also to a possible adverse effect on the patient because critical test results were not timely reported (*11*). Therefore, communication between laboratory and the clinic is extremely important, in order to obtain the necessary information about the patient in a timely manner, whether it is the test results or information from physicians for a more accurate interpretation of the test results.

This study has some limitations. First, the survey was self-reported, so participants might not have provided reliable answers. Furthermore, only the opinion of laboratory experts on interpretative comments is presented, and it is well-known that interpretative comments are the link between the laboratory and the clinic. Future studies should focus on whether the interpretative comments influence patient diagnosis and treatment, and whether interpretative comments add value to the report from physicians’ point of view.

In conclusion, although most participants state that they use interpretative comments on laboratory reports and provide expert opinions regarding test results, the need for harmonization of this part of the post-analytical phase is necessary with the purpose to establish accurate interpretation of test results. In this way, explanation about unclear test results would be provided to the clinician, which would consequently be helpful in patient diagnostic management and treatment.
